# ReHeartNet: Reconstruct Electrocardiogram From Photoplethysmography by Using Dense Connected Deep Learning Model

**DOI:** 10.1109/OJEMB.2026.3670010

**Published:** 2026-03-03

**Authors:** Shuenn-Yuh Lee, Kai-Ze Lei, Ju-Yi Chen, Chun-Rong Huang

**Affiliations:** Department of Electrical EngineeringNational Cheng Kung University 34912 Tainan 70101 Taiwan; Department of Internal Medicine, National Cheng Kung University Hospital, College of MedicineNational Cheng Kung University 34912 Tainan 70701 Taiwan; Department of Computer Science and Institute of Multimedia EngineeringNational Yang Ming Chiao Tung University 34914 Hsinchu 30010 Taiwan

**Keywords:** Cardiology, electrocardiogram, photoplethysmography, physiological signal analysis, ECG signal reconstruction, sequence-to-sequence translation

## Abstract

*Goal:* To enable comfortable and non-invasive heart rhythm monitoring, this work aims to reconstruct electrocardiogram (ECG) signals from photoplethysmogram (PPG) signals, eliminating the need for multiple electrode attachments, which are often inconvenient and may cause skin irritation. *Method:* To achieve high-fidelity ECG reconstruction from PPG inputs, we propose ReHeartNet, a novel neural network that formulates the task as a regression problem. To capture the multi-scale temporal and frequency relationships between PPG and ECG signals, the model employs densely connected bidirectional long short-term memory (DC-BiLSTM) blocks. To enhance reconstruction accuracy, hierarchical features from different BiLSTM layers are fused within the network architecture. *Results:* To validate the proposed method, experiments were conducted on four datasets: MIMIC-III, BIDMC, TBME-RR, and CBIC-Heart. ReHeartNet consistently outperforms baselines based on generative adversarial networks (GAN), recurrent neural networks (RNN), and transformers. *Conclusions:* To support reliable cardiac monitoring in various populations, ReHeartNet demonstrates strong generalization and robustness in ECG reconstruction for healthy individuals and patients with circulatory diseases and arrhythmias, using only wearable PPG signals.

## I. Introduction

Cardiovascular DISEASES (CVDs) generally describe diseases of the heart and blood vessels. According to the World Health Organization (WHO), CVDs are the leading cause of death worldwide [1]. CVD diagnosis requires long-term monitoring to capture a patient's heart rhythm using electrocardiogram (ECG) signals. Then, a doctor will diagnose CVD based on the heart rhythm recorded in the ECG signals [2], [3].

However, CVD symptoms are typically unpredictable and therefore 24-hour monitoring with an ECG Holter monitor is suggested [4]. To collect ECG signals with an ECG Holter, multiple electrodes must be attached to the patient's body [5]; this practice is uncomfortable and can cause skin allergies. Patients must also carry the instrument as they go about their daily lives. Consequently, recording ECG signals more effectively, comfortably, and economically has become an emerging issue in the medical domain.

Photoplethysmogram (PPG) signals [6], [7], [8] have been demonstrated to share some standard features with ECG signals [9], [10]. PPG signals are measured by changes in blood flow in each cardiac systolic and diastolic phase, driven by the electricity of the myocardium; ECG signals can represent them. Thus, ECG and PPG signals are highly relevant in feature structure [11], [12], [13]. Compared to an ECG Holter, PPG signal sensors are less expensive and easier to use. Thus, they are widely used in currently available wearable intelligence products, such as smartwatches or sports bracelets [7], [9], [14]. If PPG signals can be used to reconstruct ECG signals, heart rhythm can be analyzed more easily and cost-effectively [15], [16]. However, although ECG and PPG signals are sourced from the same heart pumping, there is a latency between them. In addition, the sampling frequency of PPG signals is typically lower than that of ECG signals. Therefore, reconstructing ECG signals from PPG signals is a challenging problem in the medical domain.

Recently, handcrafted feature-based ECG reconstruction methods [17] have been proposed, but they do not successfully capture the correlations between PPG signals and ECG signals. In contrast, convolution neural networks (CNNs) based methods [18], [19] and recurrent neural networks (RNNs) based methods [20], [21] have shown more promising ECG reconstruction results due to learning deep features and temporal correlations between the ECG and PPG signals. In CVDs, the interpretation of arrhythmia is often based on details of the temporal features of ECG signals. State-of-the-art deep learning methods still make it challenging to reconstruct accurate ECG signals from PPG signals for arrhythmia diagnosis.

**Fig. 1.**
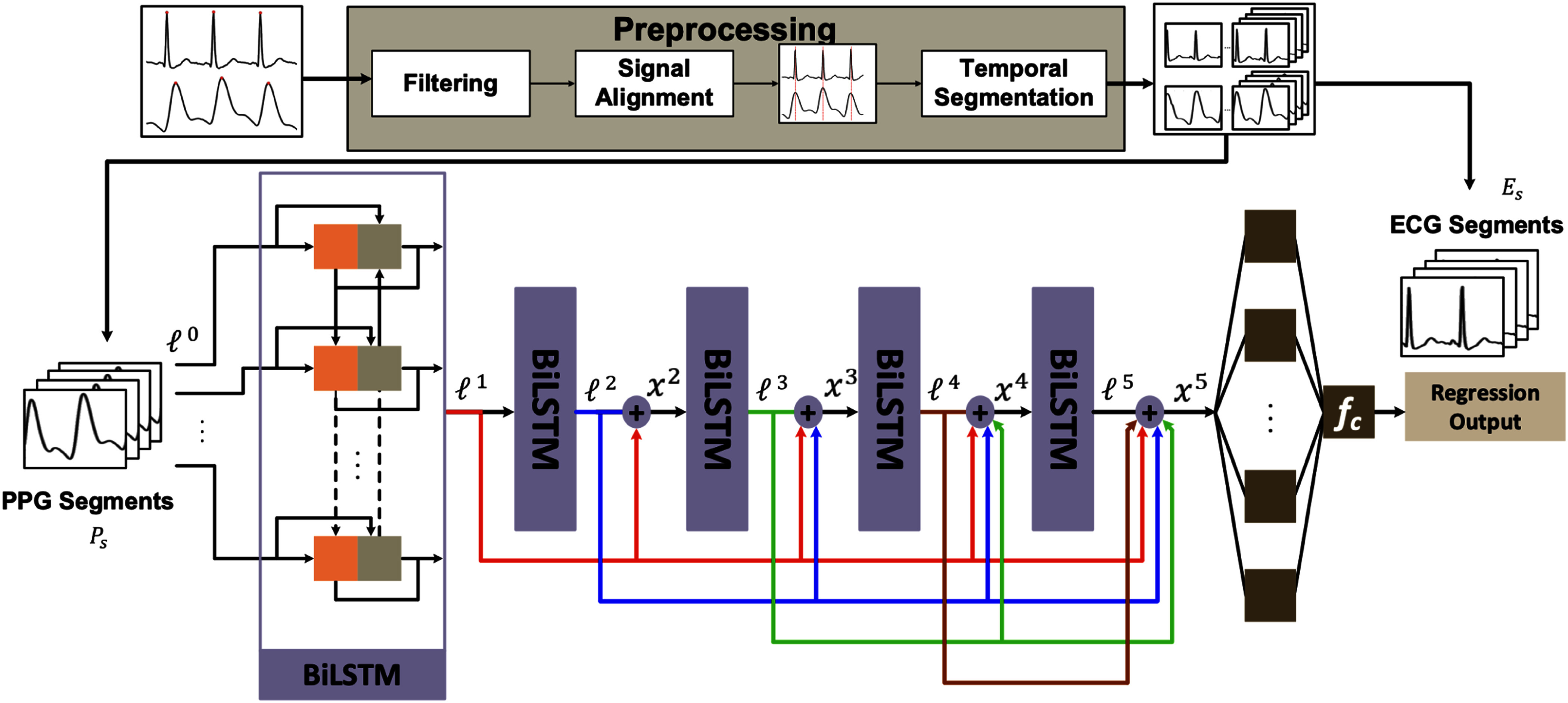
Overview of the proposed method. During training, given ECG and PPG signals, the preprocessing module is applied to obtain the corresponding temporal segments of ECG and PPG signals. Based on these temporal segments, the proposed network, which consists of a densely connected BiLSTM module, is applied to reconstruct segments of ECG signals based on the segments of PPG signals.

In the current study, we propose ReHeartNet, which comprises a preprocessing module and a densely connected bidirectional LSTM (DC-BiLSTM) module (Fig. 1), for reconstructing ECG from PPG. The former eliminates latency between ECG and PPG signals. It obtains the corresponding training segments from the input data in the training phase. At the same time, the latter learns and fuses the temporal features of various frequencies in different BiLSTM blocks to represent detailed correlations between ECG and PPG signals. The DC-BiLSTM module is built based on stacked BiLSTM blocks, and each block is densely connected to each subsequent block. In the first BiLSTM block, we aim to extract the fundamental temporal features and describe the PPG signals first. Then, dense connections are used in the subsequent BiLSTM blocks to extract detailed heart rhythm features to represent correlations between ECG and PPG signals by dispersing them in different BiLSTM blocks of the network. Moreover, our network can reduce the gradient vanishing problem [22], [23]. Detailed correlations between ECG and PPG signals can be established by combining the features of different BiLSTM blocks in the DC-BiLSTM module. Therefore, using the proposed method, an accurate heart rhythm can be reconstructed based on PPG signals.

In particular, we focus on reconstructing the lead II ECG signal, as lead II provides a clear view of most arrhythmic events and is commonly used in clinical settings for rhythm monitoring [24]. To evaluate ReHeartNet, we performed experiments on four datasets that contain the ECG and PPG signals of normal and abnormal subjects collected across different environments. The experimental results show that our method outperforms state-of-the-art methods in various metrics and datasets.

The contribution of this work is threefold. First, we adopt the hierarchical structure of the BiLSTM blocks to extract features that represent different frequencies for ECG reconstruction. Second, the proposed DC-BiLSTM module fuses features of varying frequencies from different BiLSTM blocks to represent detailed correlations between ECG and PPG signals for sequence-to-sequence ECG signal reconstruction. Finally, the proposed method outperforms state-of-the-art methods across four datasets to demonstrate its clinical applicability.

## II. Materials and Methods

Fig. 1 provides an overview of our proposed method, which is composed of two components: the preprocessing module and the DC-BiLSTM module. During training, the preprocessing module aims to remove environmental noise or motion artifacts. In addition, it aligns the ECG and PPG signals collected from different signal sources for network training. We perform temporal segmentation to divide the ECG and PPG signals based on [20]. The densely connected BiLSTM module is designed to learn representative features that capture the temporal relationships between the PPG and ECG signals. Instead of explicitly extracting predefined “heart rhythm” features, the model is trained in a data-driven manner to minimize the reconstruction error between predicted and ground-truth ECG signals. The output of the last BiLSTM block is used to reconstruct the ECG signals. The loss function minimizes the root mean square error ($RMSE$) between ground-truth ECG signals and reconstructed ECG signals based on PPG signals. It is important to note that the cardiac-phase alignment step is applied only during the training preprocessing stage to reduce systematic phase offsets and facilitate stable supervised learning. The temporal relationships between ECG and PPG signals are embedded within the model parameters. Therefore, during inference, the trained model operates without alignment between ECG and PPG signals. Instead, PPG signals are filtered with a type-I finite impulse response (FIR) filter and passed to the DC-BiLSTM module to rebuild ECG signals. The details of the modules are elaborated on below.

During training, aim to learn the correlations between the ECG and PPG segments. These two deep learning methods, including GAN and transformer model [19], [21], still require handcrafted ECG features, such as the QRS complex interval or R-R interval, to help learn transformation features specifically. However, learning handcrafted features is not feasible in clinics because the waveforms of arrhythmia are different from those of regular heart rhythms. Irregular waveforms in arrhythmia can reduce reconstruction accuracy, especially when relying on handcrafted features during learning. To address the problems mentioned above, we propose the DC-BiLSTM module to capture correlations between ECG and PPG segments without considering handcrafted features of ECG segments.

The module consists of five stacked bidirectional LSTM blocks that aim to extract hierarchical features from PPG segments and map them to ECG segments. To establish the effectiveness of hierarchical feature extraction, a naive approach is to cascade several BiLSTM blocks into a stacked BiLSTM module that learns hierarchical feature representations, compared with a single BiLSTM block. The stacked BiLSTM module can be represented as follows:

\begin{equation*}
E_{s} = \left(\ell ^{5} \circ \ell ^{4} \circ \ell ^{3} \circ \ell ^{2} \circ \ell ^{1}\right)(P_{s}), \tag{1}
\end{equation*}

where $E_{s}$ and $P_{s}$ are the $s$-th corresponding ECG and PPG segments, $\ell ^{j}$ represents the $j$-th BiLSTM block, and $\circ$ is the composition operator of the BiLSTM blocks. The output of each layer will be the input of the next layer. The stacked-BiLSTM module retrieves more temporal details of correlations between the ECG and PPG segments compared with the single BiLSTM block. Therefore, the results reconstructed by the stacked-BiLSTM module are expected to be better than those reconstructed by a single BiLSTM block. However, the features of the last layer of the stacked-BiLSTM module still make it difficult to represent the detailed waveforms of the ECG segments, as these waveforms can be considered a composition of signals at different frequencies.

Therefore, we propose using the dense connection scheme [22] to fuse the features learned in the previous BiLSTM blocks with the features of the current BiLSTM blocks to better represent the waveforms of the ECG segments. The densely connected output $x^{j}$ of the $j$-th BiLSTM block $\ell ^{j}$ is defined as follows:

\begin{equation*}
x^{j} = \sum _{k=1}^{j} \ell ^{k}, \tag{2}
\end{equation*}

where $\ell ^{k}$ represents the output vector of each BiLSTM block.

In the first BiLSTM block, we first extract the fundamental temporal features. Then, the subsequent dense connections of the four BiLSTM blocks aim to extract features of different frequencies based on the fundamental temporal features by dispersing them in different network layers, effectively learning signal components. Through dense connections, we can ensure that the subsequent layer can reuse the features of different frequencies learned in the previous BiLSTM blocks to extract more representative features between the ECG and PPG segments. In the last layer, all the features extracted from the previous layers are fused to find correlations between the ECG and PPG segments. With the learned correlations between the ECG and PPG signals, each point in the PPG segment can be reconstructed to a corresponding point in the ECG segments by a fully connected layer.

Only PPG signals are available during the inference. Therefore, PPG signals are filtered and then split into PPG segments. The segments are input into the network to obtain the reconstructed ECG segments. Then, the reconstructed ECG segments are combined to show the ECG signals of the subject.

## III. Results

### A. Datasets and Experimental Settings

In the experiments, three publicly available datasets, namely, the Medical Information Mart for Intensive Care III (MIMIC-III) [25], Beth Israel Deaconess Medical Centre (BIDMC) [26], and CapnoBase IEEE TBME respiratory rate benchmark (TBME-RR) [27] datasets, and a newly collected dataset, i.e., CBIC-Heart, are elaborated. The detailed descriptions of the publicly available datasets are provided in the supplementary material.

The CBIC-Heart dataset is a novel evaluation dataset collected by the device [28] for long-term physiological signal measurement. The device can simultaneously measure ECG and PPG signals. This dataset contains five cases of more than 30 minutes of recorded ECG and PPG signals collected in their daily life. In the experiments, we used this dataset to evaluate the accuracy and stability of each method for long-term correlations between ECG and PPG signals. It is worth noting that the four datasets have different sampling rates, which allows one to evaluate the robustness of both the proposed and baseline methods under varying temporal resolutions.

To evaluate the performance, amplitude-based metrics such as $RMSE$ and $r$ are used in [17], [18], [19], [20]. We additionally evaluate beat-timing error, which quantifies the temporal displacement of the R-peak between the reconstructed and the ground-truth ECG. ECG R-peaks in both signals are detected using the Pan–Tompkins algorithm [29], and each reconstructed ECG R-peak is matched to the temporally nearest ground-truth ECG R-peak without imposing a tolerance window. The beat-timing error is computed as the mean absolute temporal difference across all matched beats. Beat-timing accuracy is clinically relevant because ECG R-peak timing underlies heart rate estimation, HRV computation, arrhythmia detection, and the calculation of PR/QRS/QT intervals. All subject-level performance metrics are reported as mean values with corresponding 95% confidence intervals (CIs) estimated across subjects.

### B. Quantitative Results

In [19], CardioGAN uses dual generators and discriminators to reconstruct ECG signals from PPG inputs. In [20], a single BiLSTM block is used to learn the temporal mapping between PPG and ECG. The method in [21] employs a transformer-based sequence-to-sequence model [30] for signal translation. To ensure a fair and consistent comparison, we applied the source codes of CardioGAN [19] and BiLSTM [20] and re-implemented transformer [21]. Then, we trained these methods in the four evaluation datasets: MIMIC-III, BIDMC, TBME-RR, and CBIC-Heart under the same experimental settings.

Table I summarizes the performance of these methods and our proposed ReHeartNet in the four datasets using identical training protocols and evaluation metrics. Detailed training settings and hyperparameter configurations are provided in the supplementary material. The MIMIC-III dataset has the largest amount of data compared to the other three datasets to validate the models' stability in ECG signal reconstruction. In [19], temporal information in the discriminator was lacking; therefore, $RMSE$ and $r$ were relatively worse than those of the other methods. In [20] and [21], the temporal modules in the networks were considered; therefore, the performance of these methods was better than that of [19]. Based on the fusion of the features of different frequencies represented by the BiLSTM blocks, our method can successfully reconstruct ECG signals using PPG signals compared to the other methods.

The subjects in the BIDMC dataset are patients in the intensive care unit (ICU), so the waveforms of the patients' ECG signals are relatively complex compared to those of normal ECG signals. Without considering temporal information, the performance of [19] was still lower compared to that of the other methods. Although [20] considered a single BiLSTM block to learn temporal information, well reconstructing the details of the ECG signals was still tricky. The transformer model [21] is not extremely effective in extracting features from the frequency domain because the frequency domain features are complex to represent using different patches during the embedding phase. In contrast, our method combines hierarchical feature extraction and feature fusion to consider the temporal and frequency domains in feature learning and process complex ECG signals compared to the other methods. Similar situations can also be observed in the TBME-RR dataset [27].

In the CBIC-Heart dataset [28], the collected long-term ECG and PPG signals contain environmental interference and motion artifacts. This interference will affect the feature learning of the correlations between the ECG and PPG signals. Therefore, the CBIC-Heart dataset can validate the situation of ECG signal reconstruction in noisy environments. In Table I, noise interference exerts a considerable effect on the reconstruction of ECG signals, with the average of $r$ in the CBIC-Heart dataset being the lowest among all datasets. The proposed method uses dense connections to distinguish interference coverage and extract the corresponding features of ECG signals, and thus has better $RMSE$ and $r$ than the other methods.

**TABLE I:** Quantitative Results of the Different Methods in the MIMIC-III, BIDMC, TBME-RR, and CBIC-Heart Datasets

**Dataset**	**Method**	*RMSE* **(mV)$\downarrow$**	*r* ** $\uparrow$ **	**Beat-Timing Error (sec)$\downarrow$**
MIMIC-III [25]	CardioGAN [19]	1.1205 [1.1153-1.1258]	0.0394 [0.0332-0.0457]	0.3564 [0.1413-0.5715]
	BiLSTM [20]	0.1633 [0.1520-0.1746]	0.4068 [0.3637-0.4498]	0.7091 [0.2510-1.1671]
	Transformer [21]	0.1532 [0.1392-0.1672]	0.4929 [0.4489-0.5368]	0.6077 [0.2191-0.9962]
	ReHeartNet (Ours)	**0.0924** [0.0843-0.1005]	**0.8054** [0.7871-0.8238]	**0.1811** [0.0894-0.2728]
BIDMC [26]	CardioGAN [19]	0.8696 [0.8035-0.9358]	0.0421 [0.0311-0.0531]	1.3908 [1.0567-1.7249]
	BiLSTM [20]	0.1253 [0.1095-0.1411]	0.6263 [0.5730-0.6796]	0.5798 [0.5363-0.6232]
	Transformer [21]	0.1365 [0.1176-0.1553]	0.4927 [0.4131-0.5724]	5.0301 [4.6353-5.4265]
	ReHeartNet (Ours)	**0.1070** [0.0914-0.1226]	**0.7273** [0.6862-0.7684]	**0.3123** [0.2917-0.3330]
TBME-RR [27]	CardioGAN [19]	3.0960 [2.3736-3.8117]	0.0205 [0.0147-0.0262]	3.7669 [2.0306-5.5031]
	BiLSTM [20]	2.7385 [2.0752-3.4019]	0.4458 [0.3632-0.5284]	3.3304 [1.0961-5.5647]
	Transformer [21]	2.8311 [2.3946-3.2676]	0.4315 [0.3681-0.4949]	4.6809 [3.6154-5.7463]
	ReHeartNet (Ours)	**2.6575** [1.9937-3.3212]	**0.4906** [0.4024-0.5788]	**2.9507** [1.0110-4.8905]
CBIC-Heart [28]	CardioGAN [19]	1.3235 [1.3087-1.3383]	0.0121 [0.0001-0.0243]	4.7283 [3.0416-6.4149]
	BiLSTM [20]	0.1233 [0.0642-0.1824]	0.2578 [0.1162-0.3994]	4.2310 [2.2829-6.1792]
	Transformer [21]	0.1301 [0.1245-0.1357]	0.3516 [0.3018-0.4014]	7.8394 [4.8318-10.8471]
	ReHeartNet (Ours)	**0.1147** [0.1039-0.1255]	**0.4130** [0.3723-0.4537]	**0.4220** [0.2665-0.5776]

Across all four datasets, compared with the existing approaches [19], [20], [21], our method achieves the lowest beat-timing error. This improvement stems from two key design principles of ReHeartNet. First, the hierarchical BiLSTM blocks explicitly capture cross-beat temporal dependencies, enabling the model to track the systolic upstroke and ventricular depolarization more precisely. Second, our multi-scale feature fusion integrates complementary temporal and frequency-domain representations, reducing phase distortion and preventing temporal drift during reconstruction. Consequently, by preserving both the morphological fidelity and temporal alignment of reconstructed ECG signals, the proposed ReHeartNet achieves substantially better metrics compared with the other methods.

In addition, the reported 95% CIs further demonstrate the stability and robustness of the proposed method across subjects. For MIMIC-III and BIDMC datasets, ReHeartNet exhibits consistently narrower CIs for $RMSE$, $r$, and beat-timing error compared with competing methods, indicating reduced inter-subject variability and more reliable reconstruction performance. Although wider CIs are observed in TBME-RR and CBIC-Heart datasets due to increased domain shift and physiological variability, ReHeartNet maintains comparatively tighter timing-error intervals than competing methods under the strict no-tolerance evaluation protocol. We have also performed paired Wilcoxon signed-rank tests at the subject level to compare ReHeartNet against each baseline for $RMSE$, $r$, and beat-timing error. The improvements achieved by ReHeartNet were statistically significant across all datasets (all $p < 0.001$).

### C. Qualitative Results

**Fig. 2.**
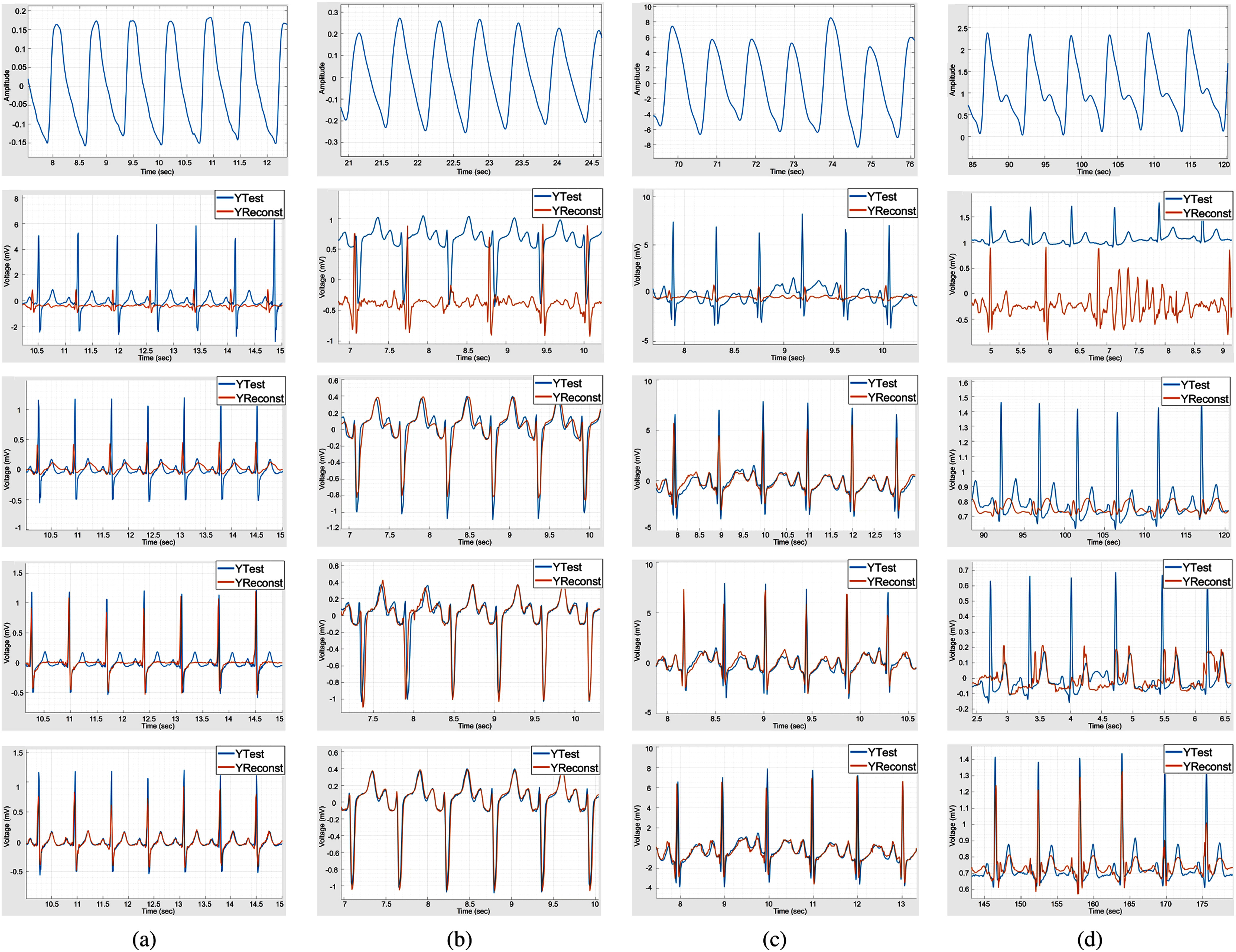
ECG reconstruction results of the (a) MIMIC-III dataset, (b) BIDMC dataset, (c) TBME-RR dataset, and (d) CBIC-Heart dataset. The first row shows the input PPG signals of the four datasets. The second to fifth rows show ECG reconstruction with respect to [19], [20], [21] and the proposed method.

Fig. 2(a) to (d) present the visualization results of the four different datasets, that is, the MIMIC-III, BIDMC, TBME-RR, and CBIC-Heart datasets, concerning [19], [20], [21], and the proposed method. The reconstruction results are shown in orange, while the ground-truth signals are shown in blue. The first row of Fig. 2 provides the original PPG signals of the subjects from the four datasets before reconstructing them into ECG signals.

Fig. 2(a) presents the ECG reconstruction results of Subject No. 2 from the MIMIC-III dataset. As shown in the second row of Fig. 2(a), the method in [19] cannot align the period of ECG signals in the waveforms, which is the most crucial feature that represents ECG signals, because temporal information is not considered in the discriminator. As shown in the third row of Fig. 2(a), the method in [20] uses a single BiLSTM block to learn temporal features and solve the alignment problem. However, the P and T waves in the waveforms are not reconstructed well because deep features in the frequency domain are difficult to extract using a single BiLSTM block. As shown in the fourth row of Fig. 2(a), the method in [21] achieves a better reconstruction of the R-peak in the waveforms compared to the method in [20]; however, the details of the ECG signals are still unsatisfactory. As shown in the fifth row of Fig. 2(a), the proposed method can extract features of different frequencies using DC-BiLSTM, and thus achieve better reconstruction results compared to the other methods in the alignment of ECG signals and the recovery of waveform details.

Fig. 2(b) presents the ECG reconstruction results of Subject No. 15 from the BIDMC dataset, whose composition of ECG signals in the frequency domain is more complex than that of normal subjects. As shown in the second row of Fig. 2(b), the method in [19] fails to reconstruct the waveforms of the ECG signals due to the alignment problem. The method in [20] considers temporal information using a single BiLSTM block. The waveform details of the reconstructed ECG signals are drifted compared with the ground-truth ECG signals, as shown in the third row of Fig. 2(b). Although the results of [21], shown in the fourth row of Fig. 2(c), reveal the potential of the transformer model in ECG reconstruction, the reconstructed ECG signals still drift. As shown in the fifth row of Fig. 2(b), the proposed method can learn temporal and complex frequency domain features by using DC-BiLSTM blocks, leading to better waveform reconstruction of ECG signals compared to the other methods.

Fig. 2(c) presents the ECG reconstruction results of Subject No. 4 from the TBME-RR dataset, in which the sensor sampling rate is higher than those of the MIMIC-III and BIDMC datasets. More data points also represent the details of ECG signals and can be reconstructed in a broader frequency band. Without considering temporal information, the method in [19] fails to reconstruct the waveforms of the ECG signals, as shown in the second row of Fig. 2(c). The third and fourth rows of Fig. 2(c) also show a drift from the ground truth because of the lack of feature representations with different frequencies. As shown in the fifth row of Fig. 2(c), the proposed method reconstructs the details of the waveforms of the ECG signals by considering the feature representations of different frequencies and the feature fusion.

Finally, the result of the ECG reconstruction of Subject No. 1 from the CBIC-Heart dataset is presented in Fig. 2(d). The collected data in the CBIC-Heart dataset are long-term and contain environmental noise and motion artifacts. Thus, the waveforms of the reconstructed ECG signals in [19], [20], [21], shown in the second, third, and fourth rows, respectively, of Fig. 2(d), drift from the waveforms of ground-truth ECG signals. As shown in the fifth row of Fig. 2(d), the proposed method can reconstruct ECG signals under environmental interference. Such results reveal the effectiveness of the proposed DC-BiLSTM module in reconstructing ECG using PPG signals.

## IV. Conclusion

In this study, we propose ReHeartNet, an effective and better solution to reconstruct ECG signals using PPG signals. The DC-BiLSTM module combines hierarchical feature extraction and feature fusion of different frequencies to learn the correlations between ECG and PPG signals. The experiments show that the regression output layer connected with the DC-BiLSTM module achieves superior performance compared with the generative adversarial network-based [19], RNN-based [20], and transformer-based [21] methods to deal with the PPG-to-ECG signal reconstruction problem in all evaluation datasets. We also discuss ECG reconstruction from the PPG signals of patients and normal subjects to demonstrate the effectiveness of the proposed method in clinics.

Currently, most of the datasets used in relevant research [31], [32], [33] have only normal and abnormal sinus rhythms. Datasets of non-sinus rhythms should also be considered for the ECG reconstruction problem. In the future, we will collect data from more CVD patients to evaluate the applicability of the proposed method in the clinical setting. Moreover, PPG signals, which are currently collected using finger fixtures, can be replaced with smartwatches to easily enjoy the benefits of heart rhythm monitoring in daily life. To enhance the robustness and generalization of our method, we will investigate the proposed method in diverse device domains and signal conditions in the future. In particular, we plan to simulate domain shifts [34], [35] by introducing wrist-worn PPG signals with realistic noise and motion artifacts, as well as to stratify performance according to signal quality indices where raw data are available.

## Supplementary Materials

The supplementary materials include details related to the loss functions and implementation details in the Materials and Methods section, the ablation study in the Results section, and the discussion.

## Conflict Of Interest

The authors have no conflict of interest to declare.

## Author Contributions

Shuenn-Yuh Lee contributed to the conceptualization and design of the methodology, refined the manuscript, and supervised the project. Kai-Ze Lei contributed to the conceptualization, design of the methodology, and network implementation, as well as performed the experimental results and the ablation study. Ju-Yi Chen contributed to the collection of clinical diagnostic data and the interpretation of the corresponding patient symptoms. Chun-Rong Huang contributed to the conceptualization and design of the methodology, refined the manuscript, and supervised the project. All authors contributed to the discussion of the results and the writing of the manuscript.
